# The influence of a priori grouping on inference of genetic clusters: simulation study and literature review of the DAPC method

**DOI:** 10.1038/s41437-020-0348-2

**Published:** 2020-08-04

**Authors:** Joshua M. Miller, Catherine I. Cullingham, Rhiannon M. Peery

**Affiliations:** 1 Department of Biological Sciences, University of Alberta, Edmonton, AB Canada; 2 Department of Biology, Carleton University, Ottawa, ON Canada

**Keywords:** Genetic variation, Population genetics, Population genetics

## Abstract

Inference of genetic clusters is a key aim of population genetics, sparking development of numerous analytical methods. Within these, there is a conceptual divide between finding de novo structure versus assessment of a priori groups. Recently developed, Discriminant Analysis of Principal Components (DAPC), combines discriminant analysis (DA) with principal component (PC) analysis. When applying DAPC, the groups used in the DA (specified a priori or described de novo) need to be carefully assessed. While DAPC has rapidly become a core technique, the sensitivity of the method to misspecification of groups and how it is being empirically applied, are unknown. To address this, we conducted a simulation study examining the influence of a priori versus de novo group designations, and a literature review of how DAPC is being applied. We found that with a priori groupings, distance between genetic clusters reflected underlying *F*_ST_. However, when migration rates were high and groups were described de novo there was considerable inaccuracy, both in terms of the number of genetic clusters suggested and placement of individuals into those clusters. Nearly all (90.1%) of 224 studies surveyed used DAPC to find de novo clusters, and for the majority (62.5%) the stated goal matched the results. However, most studies (52.3%) omit key run parameters, preventing repeatability and transparency. Therefore, we present recommendations for standard reporting of parameters used in DAPC analyses. The influence of groupings in genetic clustering is not unique to DAPC, and researchers need to consider their goal and which methods will be most appropriate.

## Introduction

Inference of genetic clusters and knowledge of their divergence and distribution are important for many aspects in evolutionary biology and population genetics including studies of speciation (Sousa and Hey 2013), inferring disease spread risk (Hampton et al. 2004; Cassirer et al. 2018), as well as applications in conservation and forensics (Funk et al. 2012; Coates et al. 2018). As such, many methods have been developed for determining genetic clusters and quantifying divergence among them. These range from admixture and Bayesian clustering analyses (e.g., STRUCTURE (Pritchard et al. 2000; Falush et al. 2003); ADMIXTURE (Alexander et al. 2009); and LEA (Frichot and François 2015)), phylogenetic approaches (Yang and Rannala 2012) and principal components analyses (PCA; Patterson et al. 2006; Reich et al. 2008), to F-statistics (Weir and Cockerham 1984) and analysis of molecular variance (AMOVA; Excoffier et al. 1992; Meirmans 2012).

Within all of these methods, there is a conceptual divide between assessing a priori (predefined) populations, versus finding clusters de novo. The former can help visualize differentiation between hypothesized groups or jurisdictions, while the latter is a test for population structure in a dataset. Both are valid questions; however, misspecification of groups can have serious consequences, especially for species of conservation concern. On the one hand, misspecification may lead to artificially large populations with Wahlund-like effects of apparent depressed heterozygosity (Wahlund 1928), and such inflated population size estimates can prevent legal protections thereby increasing the risk of extinction for one (or more) of the “cryptic” genetic clusters. On the other hand, misspecification may lead to over splitting populations that should be combined, potentially resulting in wasted resources engaging in translocations to increase population numbers, or undertaking habitat restoration to unnecessarily promote gene flow (Weeks et al. 2011; Aitken and Bemmels 2016). Commonly used methods for assessing genetic clusters differ in how they address finding de novo genetic clusters versus visualizing a priori groupings (Table 1) with some requiring a priori designations of groups (Fst, AMOVA), while others only describe de novo population structure (PCA).

**Table 1:** Conceptual breakdown of how commonly used clustering methods address finding de novo genetic clusters versus visualizing a priori groupings.

	de novo	a priori
Admixture analysis	Novel genetic clusters discovered through analysis of allele frequencies among “K” groups (Pritchard et al. 2000; Frichot et al. 2014)	Prior groupings can be specified to visualize or assist with clustering (e.g., usepopinfo flag in STRUCTURE (Hubisz et al. 2009) or supervised in ADMIXTURE (Alexander et al. 2009))
Analysis of molecular variance (AMOVA)	Novel genetic clusters are discovered through *k*-means clustering, then assessed using hierarchical F-statistics such that variance is minimized within groups but maximized among them (Meirmans 2012)	Prior groupings used to assess the proportion of molecular variance is assigned among them (Excoffier et al. 1992)
Assignment tests	N/A	Prior groupings specify known individuals from which population allele frequencies are calculated, novel individuals are then assigned to these populations based on the likelihood of their genotype in the various populations (Paetkau et al. 2004; Piry et al. 2004)
DAPC	Novel genetic clusters are discovered through *k*-means clustering then visualized via discriminant analyses (Jombart et al. 2010)	Prior groupings are taken and visualized via discriminant analyses (Jombart and Collins 2015)
F-statistics	N/A	Prior groupings used to assess the genetic distance among them (Weir and Cockerham 1984)
Phylogenetic approaches	Novel genetic clusters discovered through grouping based on sequence similarity or genetic distance among individuals	Prior genetic clusters can be specified (e.g., forced monophyly) in a series of trees and then tested against one another to see which is more statistically likely (Goldman et al. 2000)
Principal components analysis (PCA)	Novel genetic clusters discovered through eigen vector decomposition of allele frequencies among individuals (Patterson et al. 2006)	N/A

Discriminant Analysis of Principal Components (DAPC) brings together two analysis methods to assess population structure (Jombart et al. 2009, 2010; Jombart and Ahmed 2011). In this approach, implemented in the R package adegenet (Jombart 2008), multilocus genotype data are transformed using principal component (PC) analysis to derive the uncorrelated variables that serve as input for discriminant analysis (DA). The DA aims to maximize among‐group variation and minimize within‐group variation. Results are depicted as scatterplots with individuals as points, and often have inertial ellipses around groups. DAPC does not make assumption of underlying population genetic processes (e.g., linkage equilibrium, Hardy–Weinberg equilibrium) common to other methods used to detect population structure. In addition, since it is based on PC analyses, DAPC can analyze genomic datasets relatively quickly and efficiently.

While there are fewer underlying assumptions about processes of population evolution, there are key parameters that need to be carefully assessed during application of DAPC. Most importantly, the groups that will be used in the DA, as well as the number of PCs retained for DA. Groups can be defined in two ways: a priori population designations, or de novo description via clustering methods, most often *k*-means clustering within the adegenet package itself (Jombart 2008; Jombart and Ahmed 2011; Jombart and Collins 2015). Once the clusters have been defined, users must then determine the number of PCs to retain such that there is discriminatory power, but not too many such that separation between groups is inflated and individual assignment to groups becomes unstable (Jombart and Collins 2015).

Given the lack of assumptions and ability to process large datasets, DAPC has rapidly become a core technique for many population genetic studies. But as the use of DAPC has continued to rise we have noticed authors not reporting how they are applying the method to address their question of interest. This has significant implications if authors are using a priori population designations when the goal of the paper is to determine the number of genetic groups in a dataset. In addition, it is not known how robust or sensitive the method is to misspecification of the goal. To address this, we have taken a two-pronged approach: (1) a simulation study to explicitly examine at what point a priori cluster designations override lack of genetic structure in DAPC analyses; (2) a literature review of studies that used DAPC to quantify how the program is being applied, and if authors are accurately reporting their methods. We conclude by making recommendations for the parameters that should be reported in papers implementing DAPC analyses to ensure transparency and reproducibility.

## Methods

### Simulation study

We generated simulated datasets for both microsatellite and SNP loci. For both marker types we simulated two randomly mating, diploid populations with equal numbers of males and females. Simulations were run for 20,000 generations starting at the minimum diversity; we ensured the models reached stability by examining the trend-line for *F*_ST_. For the microsatellite sets we modeled genotypes at 15 loci where the mutation model was a combination of the single-step mutation (SSM: 75%), and K-allele model (KAM: 25%) with 20 variable states (*μ* = 0.001) using Easypop 1.7 (Balloux 2001). This number of loci was chosen as it was the average number seen in a previous review of papers applying STRUCTURE for determining genetic clusters (Janes et al. 2017). We created two sets of simulations differing in the starting population pools (*N*_*c*_): in the first set the two populations contained 100 individuals each, and in the second set they contained 500 individuals each. From each set, we simulated 50 replicates from five scenarios which differed in the amount of migration between populations (*m* = 0.0001, 0.001, 0.005, 0.01, 0.5), resulting in a total of 500 population replicates.

For the SNP sets we modeled genotypes at 2000 loci, representing a “genome-scale” dataset produced by reduced representation methods (e.g., Peterson et al. 2012) or a low-density SNP chip (e.g., Hagen et al. 2013; Malenfant et al. 2015). Here the mutation model was a KAM model with two variable states (*μ* = 0.0001) using Easypop 2.0.1 (Balloux 2001). In the interest of computational efficiency, we restricted our simulations to *N*_*c*_ = 500, and 50 replicates of the five migration rates (*m* = 0.0001, 0.001, 0.005, 0.01, 0.5), resulting in a total of 250 population replicates.

From each simulation replicate, of both microsatellites and SNPs, we sampled 10 individuals per population for use in DAPC analyses. DAPC analyses were conducted twice on each replicate using the R package adegenet version 2.1.1 (Jombart 2008). In the first analysis a priori population assignments were used as the population identifier. In the second analysis the find.clusters() method was used to assign samples to groups which were then used as the population identifier. To automate the assignment procedure, we used the “diffNgroup” criterion which automatically determines the “best” number of populations (*K*) based on Bayesian information criterion (BIC) differences between successive values of *K*. We tested *K* values from 1 to 10. While BIC has been shown to perform well at determining the best *K* when *K* is <5 (Verity and Nichols 2016), it is important to note that the find.clusters() method was not intended to find *K* = 1 (Jombart 2013) and our empirical experience found that using the “diffNgroup” criterion will cause the program to assign larger *K* values to unstructured datasets. Thus, we used two metrics to assess the performance of the clustering. First, the number of clusters returned, and second the accuracy of the individuals assigned to each cluster. We noted cases where: (a) the find.clusters() method assigned a *K* > 2 to a replicate, and (b) replicates where *K* = 2, but the number of individuals per cluster was different than the number sampled from each simulated population (*N* = 10). For both a priori and de novo clustering methods an initial DAPC was run considering 30 PCs, after which the optim.a.score() was used to assess the optimal number of PCs to retain. Once the optimal number of PCs was determined, a second DAPC analysis was conducted using this value. For replicates which successfully clustered (i.e. had K = 2 and 10 individuals in each group), we calculated the distance between clusters using Euclidean geometry based on the “grp.coord” values from the second DAPC analysis. For all replicates, we calculated *F*_ST_ (Weir and Cockerham 1984) between the a priori groups using hierfstat version 0.04-22 (Goudet 2005). In cases where an *F*_ST_ estimate was <0 we rounded it to 0. We used generalized linear models with a binomial error structure to examine the relationship between clustering success (dependant variable) and *F*_ST_, as well as possible covariates of marker type and *N*_*c*_. We considered three models: (1) *F*_ST_ only, (2) *F*_ST_ and marker type, and (3) *F*_ST_ and *N*_*c*_. Models were compared with the package MuMIn version 1.43.6 (Bartoń 2018) and we used AICc to assess which was the best fit. All results were visualized with ggplot2 version 3.0.0 (Wickham 2016). All analyses using R were conducted in R studio version 1.1.423 (RStudio Team 2016) using R version 3.6.0 (R Core Team 2019).

### Literature review

We searched Web of Science for all citations to Jombart et al. (2010) which describes the DAPC method (search conducted on April 17, 2019). By choosing this publication we acknowledge that this may miss authors who implement DAPC but cite the adegenet package (Jombart 2008; Jombart and Ahmed 2011) and not this method paper. However, the pool of citations generated will be representative of the greater body of literature.

From this initial pool we considered all papers from three journals that represent a broad cross-section of the publishing landscape: Molecular Ecology (a leader in the field of molecular ecology and the journal with the most papers citing the focal publication, *N* = 119), Heredity (a society journal, *N* = 22), and Ecology and Evolution (an open access publication, *N* = 65). To be included in our analyses, papers had to analyze empirical genetic or genomic data for evidence of population structure (not clustering of multigene families, e.g., MHC). From each paper that met this criterion we recorded the following information if it was present in the main text (we did not assess supplementary materials): (1) year of publication. (2) The stated goal of the analysis (i.e., finding de novo structure or visualizing a priori groups). Note that this goal was determined after reading only the abstract and introduction. (3) Did the goal match the analyses conducted (i.e., if the goal was to group samples was the find.clusters() function used)? (4) Did the authors determine the optimal number of genetic clusters in their data? (5) Did the authors explicitly state how the optimal number of clusters was chosen (i.e. find.clusters(), k-means clustering method, or report use of BIC scores)? (6) Did the authors include how the number of PCs retained was chosen (yes or no)? (7) If yes, which method of choosing PCs was used? (8) Did the authors include how many PCs were retained? (9) Were other clustering methods implemented? Here we considered three general categories: PCA, admixture (e.g. STRUCTURE (Pritchard et al. 2000; Falush et al. 2003), ADMIXTURE (Alexander et al. 2009), LEA (Frichot and François 2015), etc.), phylogeny (e.g. NJ tree), as well as an “other” category (e.g. AMOVA or isolation-by-distance analysis). (10) Were there Supplementary Materials associated with the paper. In cases where a paper analyzed multiple taxa, or multiple (sub)sets of samples or loci from the same taxa we consider these as independent “studies”.

From this database we generated summary statistics including the number of studies using DAPC for de novo structure versus visualizing a priori groups. The proportion of studies where the stated goal matched what was presented in the results, as well as if run parameters were reported. We also quantified how many studies used multiple methods for clustering genetic data. In addition, we looked for trends over time in the information reported. Specifically focusing on studies where the goal was finding de novo structure, we examined the percentage of studies published each year for the following metrics: (1) authors stated they searched for the optimal number of genetic clusters in their data, (2) authors stated the method used to determine the optimal number of PC to retain, and (3) authors stated the final number of PCs used in the DA. For each of these metrics we conducted two weighted linear regressions of percentage of studies against year, with weights corresponding to the total number of studies in that year. In the first regression, year was assessed directly as a continuous variable; while in the second, it was fit as a second-order polynomial to allow for nonlinear changes over time. The model pairs for each metric were compared with the package MuMIn as described above to assess if the second-order polynomial increased model fit. Finally, for the subset of studies which did report the method used to determine the optimal number of PCs to use in the DA we examined if there were trends in the use of specific methods. Our goal here was to see if the community has settled on a specific method. Note that three methods appeared in a single study each and therefore were not included, and for three studies which used multiple methods we added both counts to the totals of the individual method.

## Results

### Simulation study

**Fig. 1:**
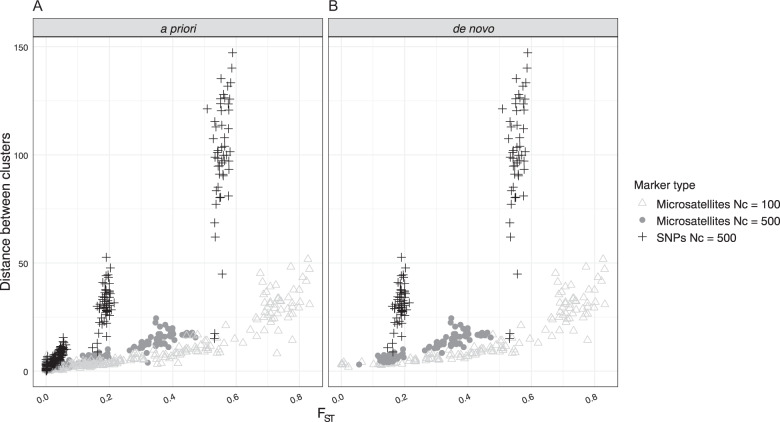
Scatterplots of Euclidean distance between DAPC clusters versus *F*_ST_ from our simulated datasets. Plots distinguish if DAPC clusters were specified a priori (**a**) or determined de novo though *k*-means clustering (**b**) as well as the marker sets within each.

When groups were specified a priori, a Euclidean distance >0 between cluster centroids was nearly always found regardless of marker type or census size. In some replicates at the highest levels of migration, the distance between groups was several orders of magnitude larger than even the largest estimate from the replicates with the lowest migration rate (e.g., 2.741 × 10^16^ versus 51.731). This was both for microsatellite replicates with *N*_*c*_ of 100 (*n* = 7) and *N*_*c*_ of 500 (*n* = 7) as well as SNP replicates (*N* = 9). Given that at high levels of migration we would expect lower distances between clusters, these outlier replicates were discarded from comparisons of the distance between clusters and *F*_ST_. For the remaining replicates, the distances between DAPC clusters decreased with increasing migration rate and were positively associated with *F*_ST_ between groups (Fig. 1a).

In contrast, when groups were not specified a priori, clusters were unsuccessfully resolved for many replicates. For both marker types and *N*_*c*_ values, clusters were successfully detected for nearly all replicates of the two lowest migration rates (95% and 91% of microsatellite replicates at *N*_*c*_ of 100 and 500, respectively, and 100% of SNP replicates). For the microsatellite replicates, where accurate clusters were not resolved (*N* = 14), the majority (*N* = 9) were due to individuals not being correctly assigned between the two clusters. However, at the three highest migration rates the vast majority of replicates were not successfully clustered (73.3% and 99.3% of microsatellite replicates at *N*_*c*_ of 100 and 500, respectively, and 100% of SNP replicates). For microsatellite replicates, the reason for unsuccessful clustering depended on the *N*_*c*_. With *N*_*c*_ = 100, misclustering events were nearly equally divided between the *k*-means clustering method suggesting that the optimal *K* > 2 (42.7%), and incorrect assignment of individuals to clusters at *K* = 2 (57.3%). In contrast, with *N*_*c*_ = 500 almost all of the misclustering was due to the *k*-means clustering identifying the optimal *K* as >2 (95.3%). For all of the SNP replicates, misclustering was due to the *k*-means clustering indicating that the optimal *K* > 2. For replicates where clusters were successfully resolved de novo, the distances between the clusters were positively correlated with *F*_ST_ between groups (Fig. 1b) as was seen when groups were specified a priori.

**Fig. 2:**
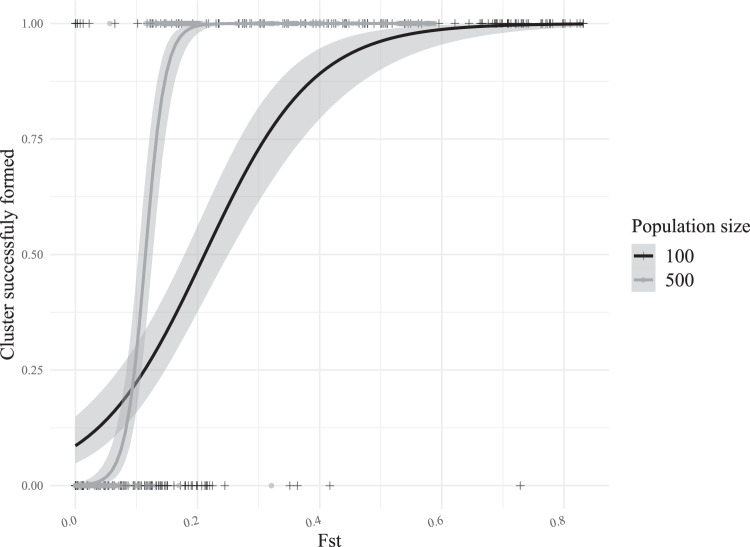
Scatter plot of relationship between *F*_ST_ from our simulated datasets and if a cluster was successfully formed by find.clusters() for either *N*_*c*_ values of 100 (black crosses) or 500 (gray circles). Curves show predictions from binomial generalized linear models for *N*_*c*_ values of 100 (black curve) or 500 (gray curve).

Our generalized linear models showed that clustering success increased with increasing *F*_ST_ between populations (Table 2), a pattern which did not significantly differ between the marker types but did differ between *N*_*c*_ values (Table 2 and Fig. 2). For *N*_*c*_ = 100, the average *F*_ST_ of successfully clustered replicates was over five times that of failed ones (0.471 versus 0.095), but the range of *F*_ST_ values for both successfully and unsuccessfully clustered replicates was very large (0.000–0.832 and 0.000–0.729, respectively). For *N*_*c*_ = 500, the average *F*_ST_ of the successful and unsuccessful replicates showed a similar disparity (0.321 versus 0.026) and range of values (0.056–0.589 and 0.000–0.321, respectively). Together, this suggests that for the demographic scenarios and sample sizes we explored the method used by find.clusters() does not reliably work when *F*_ST_ between groups is <0.1, especially for small census sizes.

**Table 2:** Results of generalized linear models examining factors associated with clustering success. Effect estimates are shown along with their standard errors.

	Intercept	*F*_ST_	Marker	*N*_*c*_	df	AICc
*F*_ST_ with *N*_*c*_	−4.78 (0.42)*	25.97 (2.02)*		1.44 (0.33)*	3	356.0
*F*_ST_ with marker type	−3.72 (0.26)*	23.88 (1.87)*	0.54 (0.29)		3	373.7
*F*_ST_ only	−3.51 (0.26)*	23.57 (1.85)*			2	375.1

*Term significant with *p* < 2 × 10^−16^.

### Literature review

Our survey of the literature resulted in a dataset of 263 studies (representing 206 publications), of which 224 studies unambiguously met the criterion for inclusion. We classified the main goal of 204 studies as finding de novo structure, 18 as visualization of a priori groups, and 2 as both. For the majority of studies, the stated goal matched what was presented in the results (140, 62.5%). While in 47 studies (21.0%) the stated goal did not match what was presented in the results and in the remaining 37 (16.5%) studies it was unclear if the goals matched the results presented. This lack of clarity often came from omission of key run parameters. In studies where the primary goal was finding de novo structure, only 39.3% (*N* = 81) stated that the optimal *K* value was found using the find.clusters() command, *k*-means clustering method, or report of a BIC score. Similarly, other run parameters needed to ensure repeatability of the analyses were often not presented. Across all studies considered, less than half (47.7%, *N* = 107) reported the method by which the optimal number of PCs for DA were retained. Even fewer studies (*N* = 78) reported the final number of PCs. Given the large proportion of studies missing information, we examined the supplementary materials for 40 studies with missing run parameters and contained supplementary materials. This ensured that our choice to focus on the main text of papers did not bias our results. We found only three studies containing relevant additional information in their supplementary materials. Therefore, it does not appear that details of DAPC analyses were placed in supplemental material and therefore missed by our review. We also reduced our dataset to one data-point per publication to assess if pseudo-replication at the level of publication was driving the patterns we observed. This reduced dataset showed similar patterns for: whether the stated goal matched analyses presented (59%, *N* = 100), indicating how the optimal *K* value was found (35.5%, *N* = 54), and reporting the method by which the optimal number of PCs were selected (51.3%, *N* = 73).

The vast majority of studies used at least one additional clustering method to assess population structure (204, 91.1%). Among the four broad categories we considered (PCA, admixture analysis, phylogeny, and other) 35.3% of studies used two methods, and 22.0% of studies used three or more methods.

**Fig. 3:**
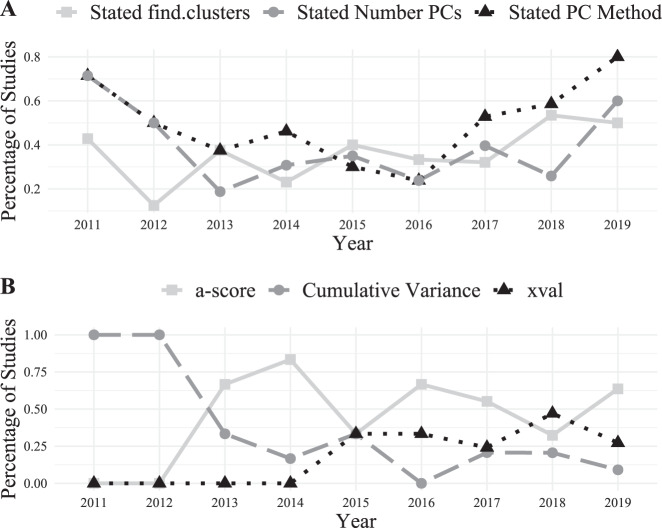
Temporal trends in reported parameters from our literature review of studies using the DAPC method. **a** Trends in the yearly proportion of studies reporting if the authors stated their method for determining the optimal number of clusters (solid line with squares), the method used to determine the optimal number of PCs to retain (dotted line with triangles), and reporting the final number of PCs retained (dashed line with circles). **b** Trends in the yearly proportion of studies reporting use of either the a-score (solid line with squares), xval (dotted line with triangles), or cumulative variance (dashed line with circles) approach to determine the optimal number of PCs to retain.

The percentage of studies reporting that the authors searched for the optimal number of genetic clusters in their data and the percentage stating the final number of PCs used when conducting DAPC analyses has remained essentially flat for the period of time we consider (2011 through April 2019; Fig. 3a). For these two metrics, fitting year as linear rather than polynomial was chosen, but the effect was not significant (optimal number of clusters: *F*_1,7_ = 3.78, *p* = 0.10; number of PCs *F*_1,7_ = 0.214, *p* = 0.66). In contrast, a second-order polynomial was a better fit to the change over time in the proportion of studies stating the method used to determine the optimal number of PCs to retain (*F*_2,6_ = 11.86, *p* = 0.008). Specifically, there was initially a very high proportion of studies reporting the PC selection method, but this decreased from 2011 to 2016. Whereas since 2016 the proportion of studies reporting their PC selection method has increased steadily, reaching a high of 80% in 2019.

Only three methods for determining the optimal number of PCs to retain met our criterion for analysis: cross-validation (xval), a-score, and cumulative variance. The cumulative variance approach was the only method used in 2011 and 2012. However, since 2012 its use has decreased (Fig. 3b). Use of xval did not start until 2015 (corresponding with release of adegenet 2.0 and its associated tutorial (Jombart and Collins 2015)); and since then, has had relatively steady application (between 33 and 47% of studies). Similarly, the a-score method was not applied until 2013 where it represented 67% of uses, however since 2015 its application has been on par with the xval method ranging between 32 and 67%.

## Discussion

With the continued increase in application of DAPC to assess population genetic structure we set out to examine the sensitivity of the method to recover (lack of) genetic clusters over a range of migration rates, for both microsatellite datasets and genomic SNPs, as well as the influence of whether or not groups were specified a priori. In addition, we surveyed the literature to examine how authors are reporting their use of DAPC.

When groups are specified a priori, find.clusters() will nearly always return a distance between the specified groups, regardless of marker type or original census size. Encouragingly, we found that this distance is correlated to underlying *F*_ST_. The ability of the method to describe structure over a wide range of differentiation is perhaps not surprising given the DA is meant to maximize among group differentiation. However, it has obvious implications for how authors assess the presence of genetic clusters in their data, and compels transparent reporting of how groups were defined in order for the research community to assess their validity.

In contrast, when groups are searched for de novo, find.clusters() has highly variable success. At low migration rates accuracy is high and distance between clusters continues to reflect underlying differentiation. At higher migration rates there is considerable inaccuracy, both in terms of the number of genetic clusters suggested, and placement of individuals into those clusters. Our simulations highlighted that for the migration scenarios and sample sizes we used, the breakdown of this method began when *F*_ST_ values were <0.1 (migration rate = 0.005). However, similar to what has been done for other clustering methods (Latch et al. 2006; Patterson et al. 2006; Janes et al. 2017; Cullingham et al. 2020), exploration of more migration scenarios with different numbers of sampled individuals and loci will be needed to firmly establish a detection threshold. The likelihood of inaccurate clustering was dependant on underlying census size, not marker type, with larger populations showing a starker transition in differentiation between successful and unsuccessful replicates. The majority of failures were due to unsuccessful selection of *K* = 2, suggesting that the method will miss-assign or not detect populations in the face of low levels of differentiation. This has implications for finding subtle structure (e.g., on small spatial scales (Benestan et al. 2015; Viengkone et al. 2016)) as well as for use in organisms with naturally high migration rates (e.g., wind pollinated plants, broadcast spawners).

By necessity we did not look at the BIC or scatterplots resulting from our simulations, but inspection of these may give more evidence for (lack of) structure regardless of how groups were specified (Box 1). It is also important to bear in mind that our sampling was even and limited to ten individuals per population. Future studies could investigate how accuracy may differ when more individuals are sampled per population, or when sampling is uneven between genetic groups. The latter of which has been shown to influence the results of other genetic clustering methods (Puechmaille 2016; Wang 2017).

The influence of a priori groupings on recovery of biologically meaningful clusters is not unique to DAPC. Other methods relying on a priori group designations (e.g., *F*_ST_, AMOVA) will similarly return values for between group differentiation in the face of “effective panmixia.” Often though, these analyses will provide a measure of significance for the estimate (Excoffier et al. 1992). Similarly, in a phylogenetics analysis, constraining topologies to represent different a priori groups can be enforced, with the “best” relationship among individuals/groups assessed via the approximately unbiased test (Shimodaira 2002) or other tree comparison method (Goldman et al. 2000). In Bayesian admixture analyses predefined populations can be used to help detect “subtle population structure” (Hubisz et al. 2009; Alexander et al. 2009) and multiple methods have been developed to assess the validity of different clustering solutions (e.g., Evanno et al. 2005; Puechmaille 2016). It is important to remember that use of a priori groups is necessary for estimating migration rates among locations (Yamamichi and Innan 2012) as well as implementation of assignment tests (Paetkau et al. 2004), which are key for forensic applications (Manel et al. 2002; Ogden and Linacre 2015).

Determining the number of genetic clusters in a dataset de novo is a nontrivial task. Our review of the literature suggests that such analyses are more common than investigating predefined groups. As such, a number of programs have been developed to address this goal (Table 1), with associated methods for assessing the validity of the clusters. However, all methods for determining de novo genetic structure will face detection limits when differentiation between groups is low. For instance, Latch et al. (2006) found that STRUCTURE did not accurately detect populations when *F*_ST_ was <0.03. While in their work describing PCA as a tool for detecting population structure Patterson et al. (2006) showed that the ability to differentiate groups with this method depends on the number of markers and individuals considered such that populations will not be detected when *F*_ST_ < $\frac{1}{{\sqrt {N_M \times N_{{\mathrm{ind}}}} }}$, where *N*_*M*_ is the number of markers and *N*_ind_ is the number of individuals genotyped. For the SNP sets in our study this *F*_ST_ should have been 0.005. In addition, assessing lack of population structure has remained an issue in population genetic studies (Janes et al. 2017; Cullingham et al. 2020). Thus, we urge researchers to think carefully about which methods are appropriate for the specific question they are addressing and explicitly state when a priori groups are enforced.

Our review of the literature showed that the majority of studies use DAPC for finding de novo structure and, encouragingly, we found that the stated goal often matches the methods and results. However, there were still a nontrivial number of studies where the stated goal did not match the reported results (21.0%), and a similar proportion where it was not clear if the methods and stated goal matched (16.5%). In addition, across all studies a number of key run parameters were not being reported. In particular, how the optimal number of genetic clusters was determined, how the optimal number of PCs wereas determined, and what that final number of PCs retained was. This final parameter is especially troubling as determination of the optimal number of PCs is necessary to avoid overfitting of the data and creating artificially large separation between groups (Jombart and Collins 2015). This lack of reporting has not changed over time, despite the increased use of DAPC. For studies that did report the method used for determining the optimal number of PCs retained, the xval and a-score procedures are used at about equal frequency suggesting that there is no clear standard operating procedure. Finally, we found that almost all studies that used DAPC applied at least one additional method for clustering genetic data, most often a Bayesian admixture analysis. So, while we are focusing on DAPC here, in practice researchers are not relying solely on this method.

Accurate and thorough reporting of run parameters, along with archiving of raw data, are essential to ensure repeatability and transparency of research. While detailing these parameters can seem burdensome when researchers are faced with page limits, such practices have become standard for many comparable methods. An apt parallel is with the program STRUCTURE where almost all papers now routinely report key information for repeatability of STRUCTURE runs (e.g., number of MCMC iterations, number of genetic clusters [*K*] explored, number of repetitions of each *K*, and how optimal *K* was selected). This reporting has likely been spurred after a period where best practices were developed and discussed in the literature (Pritchard et al. 2000; Evanno et al. 2005; Gilbert et al. 2012; Puechmaille 2016; Janes et al. 2017; Wang 2017; Cullingham et al. 2020). Therefore, it may be that standard reporting metrics have not been crystallized for the relatively newer DAPC method. To help the research community develop this standard reporting we present recommendations for documenting parameters used in DAPC analyses in Box 2.

As new methods for determining the number of genetic clusters in a dataset are continuously being introduced (e.g., Bradburd et al. 2018; Wang 2019) and best practices for others refined (e.g., Gilbert et al. 2012; Verity and Nichols 2016; Janes et al. 2017; Cullingham et al. 2020) researchers are turning to a “total evidence approach,” using multiple analysis methods on their data. In this midst of such analyses, it is important to step back and assess assumptions underlying these methods as well as our ultimate goals when applying them (Meirmans 2015; Allendorf 2017). Here we have highlighted the conceptual divide between assessing predefined populations versus finding novel clusters, and how this can influence the results of one such clustering method. Clearly, both visualization of a priori groups and de novo discovery are important, valid goals in population genetics. However, our results emphasize the need for researchers to be transparent in stating both their goal, and the precise methods used to achieve them.

Box 1: Exemplar DAPC analyses Here we present a series of analyses on two of our simulated SNP datasets. Plots labeled A correspond to a simulation with a migration rate of 0.001 (*F*_ST_ = 0.16), plots labeled B to a simulation with a migration rate of 0.01 (*F*_ST_ = 0.02).

**PCA**

In these scatterplots we can see that samples from each population can be differentiated in both datasets, with distance between clusters along PCl reflecting differentiation between populations. Some intrapopulation variation is seen on PC2.
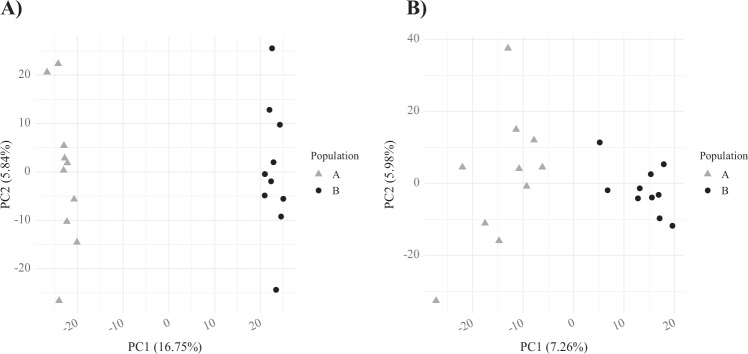

**DAPC with a priori groups**

In this case, density plots (rather than scatterplots) are shown as the majority of variation is present on one PC. Again, samples from each population can be differentiated in both datasets with distance between clusters along the *x*-axis l reflecting differentiation between populations.
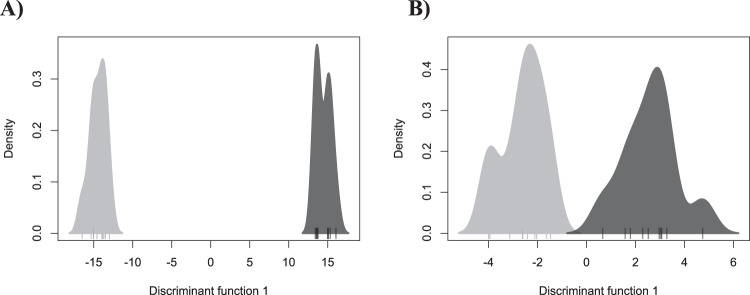

**BIC plots from de novo DAPC clustering**

When assessing de novo population structure with DAPC the optimal number of genetic groups (*K*) is often determined as that with the lowest BIC values from find.clusters(). In A we see a clear “elbow” pattern with the lowest BIC value at *K* = 2. In B there is no elbow, rather the BIC values continuously increase from *K* = 1. This pattern may cause researchers to suggest that *K* = 1, leading to under-splitting of differentiated groups. Alternatively, researchers could select *K* = 2, but when examining empirical datasets they would not be able to assess the accuracy of these groups or the individuals placed into them.
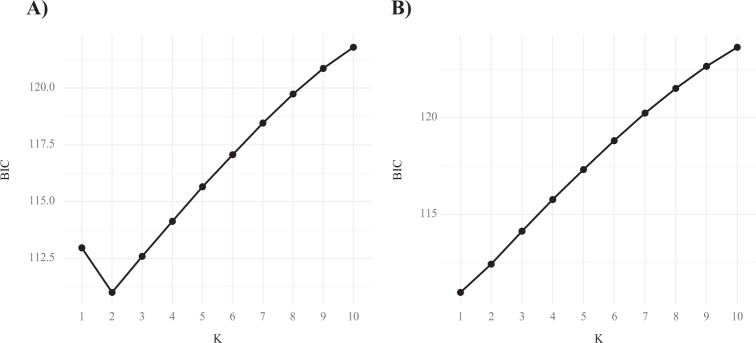

**DAPC with de novo groups**

The optimal number of groups determined above are then used for DAPC. With the stronger differentiation seen in the first simulation, plot A is essentially identical to that produced with a priori groups. In contrast, with groupings selected by find.clusters() and using the “diffNgroup” criterion (*K* = 10), plot B shows multiple groups with no connection to the simulated populations.
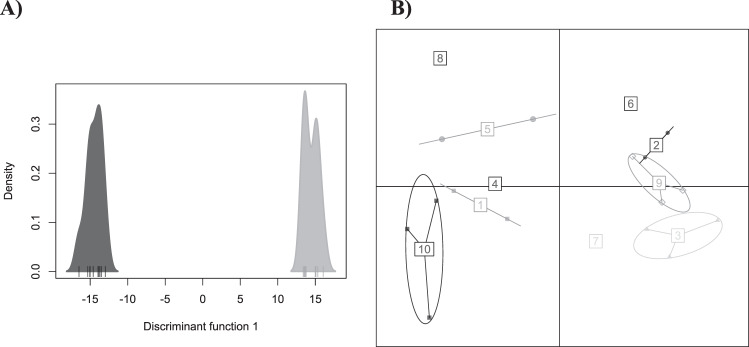

Box 2: Recommended standard reporting for DAPC analyses Our literature review of 263 empirical studies showed that many did not report run parameters necessary for transparency and rateability of analyses. In addition, based on those studies which did report such parameters, it was clear that no “standard operating procedure” has crystalized among researchers applying this method. Therefore, we developed the following list of parameters which should be reported in all DAPC analyses:

1. Explicitly state the clusters or clustering method used: were groups defined a priori or determined de novo using find.clusters()?
2. State how optimal number of *K* was chosen: when find.clusters() is used, how was the optimal number of clusters (*K*) chosen (e.g., lowest point of BIC graph or automated detection)?
3. Include documentation for selection of *K*: when using BIC, include the BIC plot or values for each *K*.
4. State the method used to determine how many PCs to retain: often a-score or xval; given that there is no “preferred” method when determining the number of PCs retained, including this data this is especially important for repeatability.
5. State the final number of PCs applied: this can appear either in the main text or figure legend showing the DAPC plot; inclusion of these values is essential for repeatability of the results presented.

**Example of minimum adequate reporting**

Methods: DAPC analyses were conducted twice to examine the influence of a priori groupings on the results. In the first analysis, sampling locations were used as a priori groups. In the second analysis, the find.clusters() function was used to determine the number of groups (*K*) de novo, with optimal *K* selected as that with the lowest BIC value. For both analyses, the optimal number of PCs to use in the DAPC was determined using the optim.a.score() command.

Results: when sampling locations were used as a priori groups, the optimal number of PCs retained were Y. Without predefined groups, the optimal *K* was found to be *W* (see Supplementary Fig. Q for BIC plot), and the optimal number of PCs retained for analysis were *Z*.

## Data archiving

The R script used to conduct DAPC analyses, measure *F*_ST_, and conduct regression analyses in the simulation study along with the database of papers used in the literature review and associated statistics have been deposited in Dryad 10.5061/dryad.4tmpg4f76.
